# Alterations of mesenchymal stem cells on regulating Th17 and Treg differentiation in severe aplastic anemia

**DOI:** 10.18632/aging.204500

**Published:** 2023-01-30

**Authors:** Ju-Pi Li, Kang-Hsi Wu, Wan-Ru Chao, Yi-Ju Lee, Shun-Fa Yang, Yu-Hua Chao

**Affiliations:** 1 Department of Pathology, School of Medicine, Chung Shan Medical University, Taichung, Taiwan; 2 Department of Pediatrics, Chung Shan Medical University Hospital, Taichung, Taiwan; 3 Department of Pediatrics, School of Medicine, Chung Shan Medical University, Taichung, Taiwan; 4 Department of Pathology, Chung Shan Medical University Hospital, Taichung, Taiwan; 5 Institute of Medicine, Chung Shan Medical University, Taichung, Taiwan; 6 Department of Medical Research, Chung Shan Medical University Hospital, Taichung, Taiwan; 7 Department of Clinical Pathology, Chung Shan Medical University Hospital, Taichung, Taiwan

**Keywords:** aplastic anemia, bone marrow failure, immunomodulation, mesenchymal stem cells

## Abstract

Immune-mediated hematopoietic destruction is a key factor in idiopathic severe aplastic anemia (SAA). With great immunomodulatory functions, mesenchymal stem cells (MSCs) are important for bone marrow niche. While the underlying etiology of immunologic changes in SAA bone marrow remains unknown, dysfunctional MSCs are implicated as a major cause. To provide evidence for their defects in immunomodulation, alterations of SAA MSCs in regulating T cell differentiation were determined. During differentiation from CD4+ T cells into T helper 17 (Th17) cells under polarization conditions, impaired inhibition on IL-17 and IL-1β production was noted when cocultured with SAA MSCs compared to control MSCs (*P* < 0.05). After stimulation of Th17 activation, the percentage of IL-17-secreting cells was significantly increased in the SAA group (9.1 ± 1.5% vs 6.6 ± 0.4%, *P* < 0.01). Under regulatory T (Treg) polarization, a higher percentage of CD4+CD25+FoxP3+ Treg cells was detected when cocultured with SAA MSCs compared to control MSCs (8.1 ± 0.5% vs 5.8 ± 0.8%, *P* < 0.01). Inconsistently, transforming growth factor-β (TGF-β) concentrations in the culture supernatant were decreased and IL-1β concentrations were elevated in the SAA group. Our data indicated impaired inhibition of SAA MSCs on Th17 activation and aberrant regulation of SAA MSCs on Treg differentiation. Increased IL-17 and IL-1β levels with decreased TGF-β levels in the supernatant suggested the potential of SAA MSCs for triggering a hyperinflammatory environment. Dysfunctional MSCs could contribute to the lack of immunoprotection in the bone marrow, which may be associated with SAA.

## INTRODUCTION

Childhood severe aplastic anemia (SAA), which is a paradigm of bone marrow failure syndromes, is a rare but potentially life-threatening disease with an annual incidence of 1-6 per million [1–3]. In most children, a specific cause cannot be identified for SAA, which has been termed “idiopathic SAA”. Although the etiology of idiopathic SAA remains to be elucidated, the most common accepted mechanism is bone marrow damage by a dysregulated immune system leading to increased apoptosis in hematopoietic stem and progenitor cells [4].

Mesenchymal stem cells (MSCs), firstly isolated from the bone marrow [5], play an important role in establishing the specialized bone marrow microenvironment for survival and differentiation of hematopoietic stem cells (HSCs). Having the activity known as a sensor and switcher of the immune system, MSCs could enhance inflammation while the immune system is underactivated and restrain inflammation when the immune system is overactivated [6]. Therefore, dysfunctional bone marrow MSCs may result in a chaotic immune status in the bone marrow niche and predispose to hematopoietic impairment, that is the characteristic of SAA.

In our previous work, MSCs were co-transplanted during HSC transplantation with faster HSC engraftment in two patients with SAA [7], suggesting the possibility of MSC insufficiency in SAA. Our previous studies demonstrated abnormalities of SAA MSCs in proliferation, differentiation, hematopoietic support, and gene expression [8–10]. Alterant T cell subsets and associated cytokine levels have been reported to play a key pathogenic role in SAA [11], and a major proportion of patients respond well to T-cell directed immunosuppressive therapy, such as antithymocyte globulin [12]. Therefore, the present study aimed to additional evidence for SAA MSCs insufficiency, focusing on their influence upon T cell differentiation.

## RESULTS

### Patients

Five patients with SAA and five controls were enrolled in this study. Clinical data of these patients were outlined in Table 1, and all bone marrow aspirates were collected at the time of diagnosis without concomitant medication. All patients were previously untreated and aged less than 18 years old. The average ages of SAA patients and controls were 12.5 and 13.7 years, respectively. For SAA patients, chromosome breakage analysis, flow cytometry, and cytogenetic studies were done to screen for Fanconi anemia, paroxysmal nocturnal hemoglobinuria, and myelodysplastic syndrome. Secondary aplastic anemia following drugs, toxic exposure, autoimmune disorders, and infections was excluded. In all of the five patients with SAA, no specific cause of pancytopenia and bone marrow failure was identified.

**Table 1:** Clinical data of the patients.

**Patient no.**	**Gender**	**Age (years)**	**Diagnosis**
**Five patients with SAA**		
A1	Male	17.1	Idiopathic SAA
A2	Male	9.3	Idiopathic SAA
A3	Female	13.4	Idiopathic SAA
A4	Male	10.3	Idiopathic SAA
A5	Female	12.2	Idiopathic SAA
**Five controls**		
C1	Male	16.3	Stage I rhabdomyosarcoma
C2	Female	5.9	Stage I Ewing’s sarcoma
C3	Female	15	Stage I rhabdomyosarcoma
C4	Male	13.5	Systemic lupus erythematosus
C5	Male	17.8	Kikuchi disease

SAA, severe aplastic anemia.

### Characterization of MSCs

**Figure 1:**
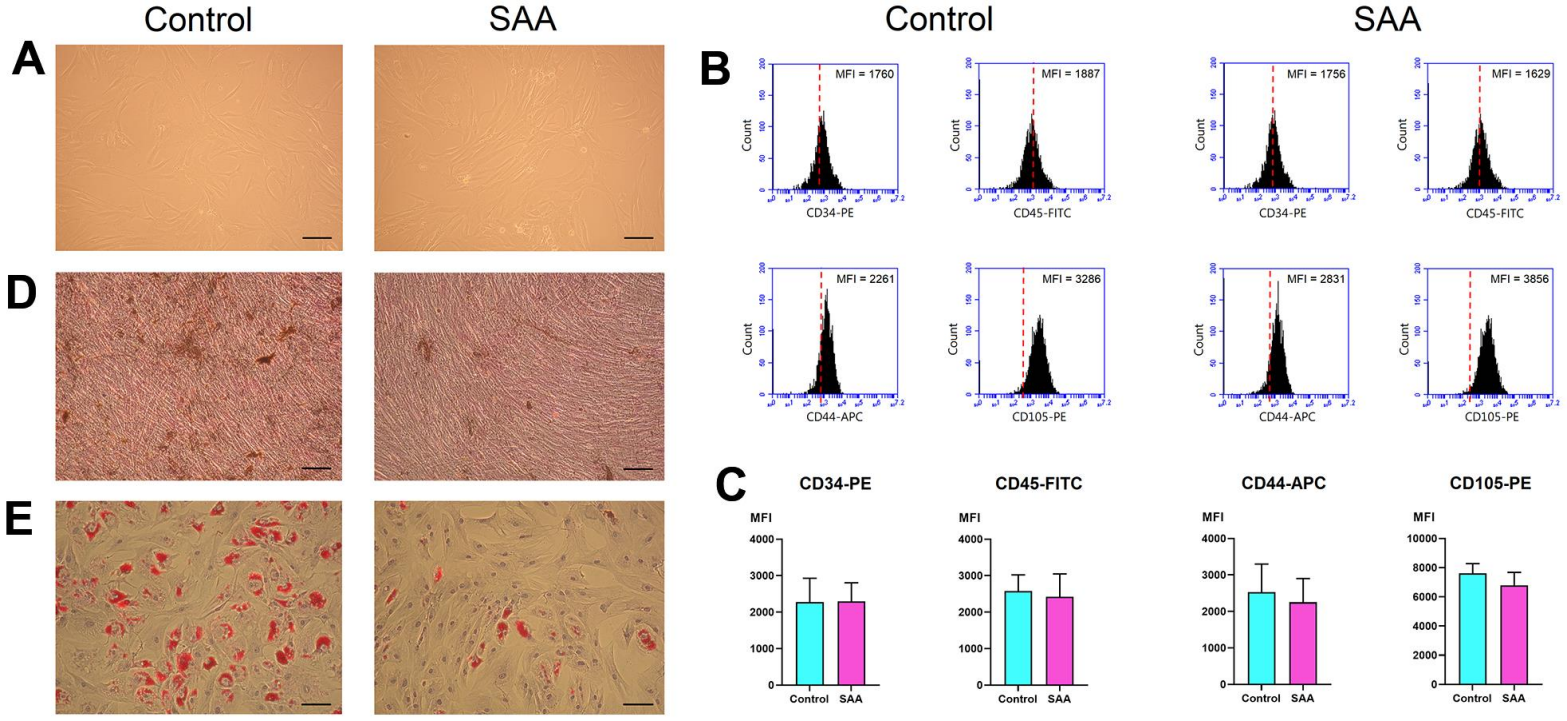
**Characterization of MSCs.** (**A**) *In vitro* culture, MSCs in the control and SAA groups shared similar growth patterns and morphologies (×100; scale bar = 100 μm). (**B**) Using flow cytometry, these cells were negative for CD34 and CD45, and positive for CD44 and CD105. The red dashed line indicates the isotype control; the black area indicates the stained cells. (**C**) There was no significant difference in the expressed MFI of CD34-PE, CD45-FITC, CD44-APC, and CD105-PE between the SAA group and the control group. (**D**) Osteogenic differentiation was demonstrated by mineralized deposits stainable with von Kossa stain (×100; scale bar = 100 μm). (**E**) Adipogenic differentiation was confirmed by intracellular accumulation of lipid droplets stainable with oil red O (×100; scale bar = 100 μm). MFI: Mean fluorescence intensity; ns: not significant; SAA: Severe aplastic anemia.

*In vitro*, MSCs displayed fibroblast-like morphologies and adhered to plates when maintained in culture conditions (Figure 1A). There was no obvious difference in cell size and shape between MSCs from patients with SAA and those from controls. MSCs revealed a consistent immunophenotypic profile which was negative for CD34 and CD45, and positive for CD44 and CD105 (Figure 1B and Supplementary Figure 1). No significant difference was noted in the expression of any single surface marker between the two groups, as shown by the detected mean fluorescence intensity (Figure 1C). MSCs in both groups could achieve osteogenesis and adipogenesis when exposed to differentiation induction medium. However, MSCs from patients with SAA had less robust osteogenic differentiation, compared to those from controls. Less extent of mineralization in the SAA group was demonstrated by less intense von Kossa staining (Figure 1D). SAA MSCs also gave rise to less lipid-containing cells after adipogenic induction. The intracytoplasmic droplets of neutral fat stained by Oil red O were less and smaller within a single adipocyte in the SAA group (Figure 1E). Nevertheless, these findings fulfilled the criteria of International Society for Cellular Therapy to define human MSCs [13].

### Purification of isolated CD4+ T cells

CD4+ T cells were obtained from splenocytes of C57BL/6 mice. Using flow cytometry analysis, about 32.9% of splenocytes were CD4+ cells (Supplementary Figure 2A). Purified CD4+ cells were further isolated by negative selection, and the final purity was 94.9% (Supplementary Figure 2B). The purified cells were used as CD4+ T cells for the following experiments.

### Impaired inhibitory effects of SAA MSCs on IL-17 and IL-1β production during Th17 differentiation

**Figure 2:**
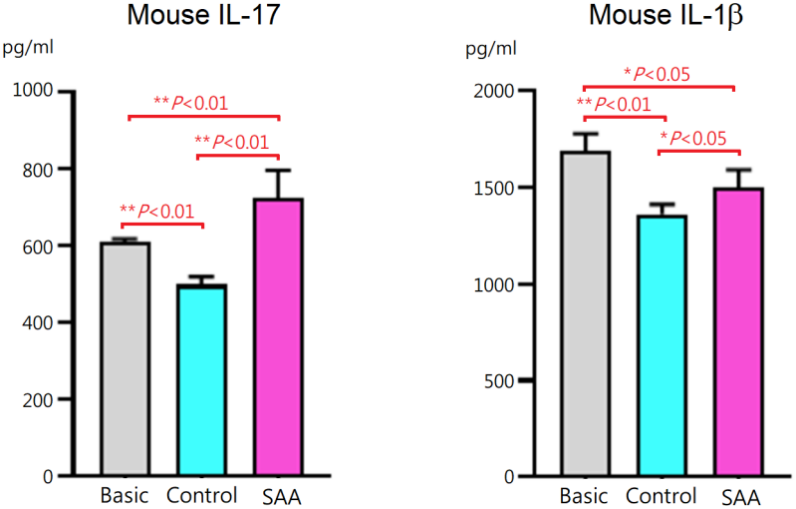
**IL-17 and IL-1β concentrations in the culture supernatant after 5-days Th17 differentiation induction.** IL-17 and IL-1β levels were lower when CD4+ T cells cocultured with control MSCs. The inhibitory effects were significantly decreased when cocultured with SAA MSCs. Data are presented as the mean ± SD. *n* = 5 in each group.

To assess effects of MSCs on T helper 17 (Th17) differentiation, mouse CD4+ T cells were cocultured for 3 days with control MSCs or SAA MSCs after 48 hours of differentiation induction. At the end of differentiation on day 5, concentrations of mouse IL-17 and IL-1β in the cell culture supernatant were measured. Compared to the basic group, IL-17 and IL-1β levels in the supernatant were lower in the control group (*P* < 0.01), suggesting that coculture of CD4+ T cells with control MSCs inhibited IL-17 and IL-1β secretion during the process of differentiation into Th17 cells (Figure 2). These data suggested that MSCs of human origin could express immunosuppressive activities on immune cells derived from mouse splenocytes. It is interesting to note that IL-17 and IL-1β levels in the SAA group were significantly increased compared to the control group (*P* < 0.01 and *P* < 0.05, respectively). These results indicated that inhibitory effects of SAA MSCs on the production of IL-17 and IL-1β during Th17 differentiation were attenuated.

### Impaired inhibition of SAA MSCs on Th17 activation

**Figure 3:**
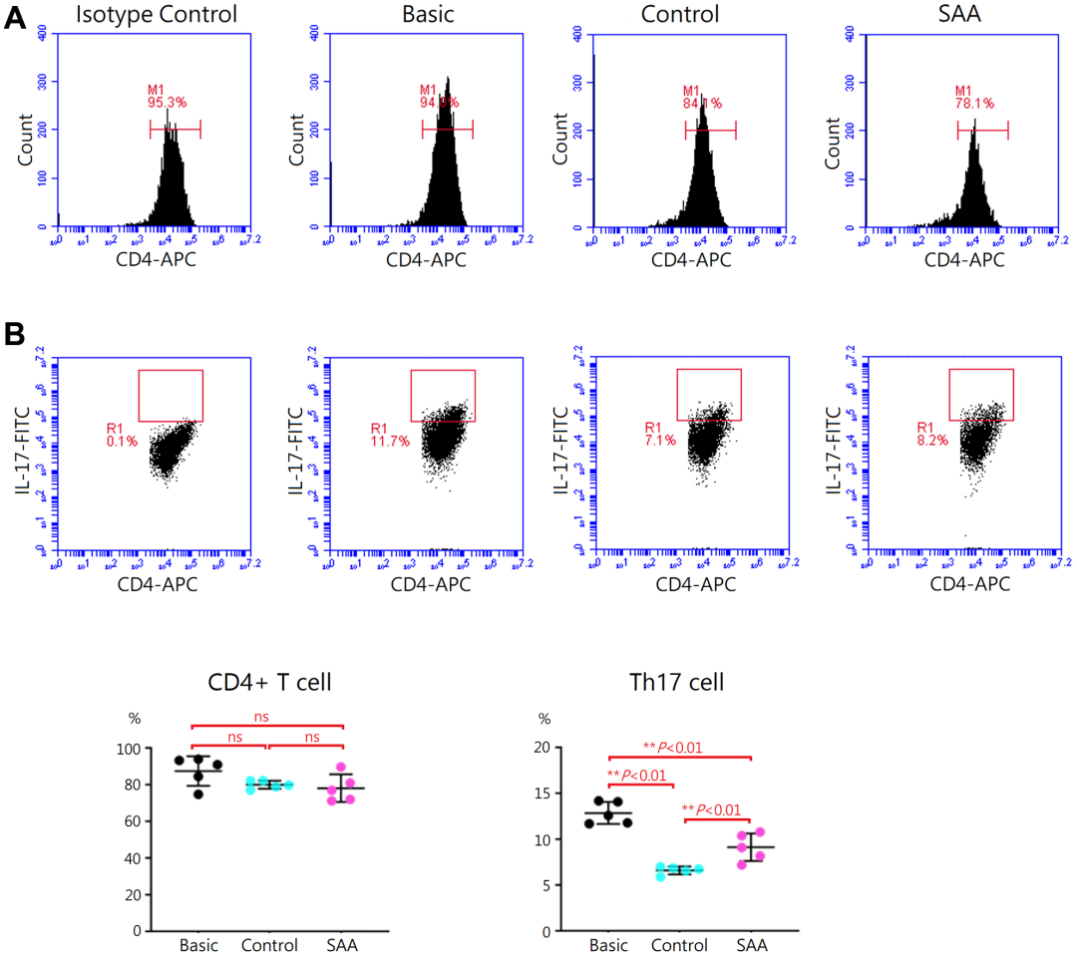
**Assessment of Th17 activation after 5-days Th17 differentiation.** (**A**) Activation of Th17 cells was confirmed by intracellular staining for IL-17 with flow cytometry. (**B**) The percentage of IL-17-secreting cells were lower in the control group, compared to the basic group (6.6 ± 0.4% vs 12.9 ± 1.2%, *P* < 0.01). The percentage of IL-17-secreting cells were significant higher in the SAA group, compared to the control group (9.1 ± 1.5% vs 6.6 ± 0.4%, *P* < 0.01). Data are presented as the mean ± SD. *n* = 5 in each group.

Following 5-days Th17 differentiation, the cells were activated with phorbol 12-myristate-13 acetate and ionomycin. As shown in Supplementary Figure 3, mouse IL-17 and IL-1β in the supernatant were at similar levels in the basic, control, and SAA groups. It was explained by that cytokines produced by the activated cells were captured in the rough endoplasmic reticulum and Golgi apparatus by the protein transport inhibitor, Brefeldin A. Activation of Th17 cells was confirmed by intracellular staining for IL-17 with flow cytometry (Figure 3A). Compared to the basic group, there was no obvious change in the percentage of CD4+ T cells either in the control group or SAA group after the stimulation of the Cell Activation Cocktail, implicating that coculturing with MSCs did not affect the quantity of CD4+ T cell population (Figure 3B). Further analysis showed that a population of IL-17-secreting cells was documented in the basic group at the percentage of 12.9 ± 1.2% under Th17 skewing conditions. In the control group, the percentage of IL-17-secreting cells was significantly decreased (6.6 ± 0.4%, *P* < 0.01), suggesting that coculturing with control MSCs could inhibit functional activities of Th17 cells. Compared to the control group, the percentage of IL-17-secreting cells was significantly increased in the SAA group (9.1 ± 1.5%, *P* < 0.01), indicating impaired inhibition of SAA MSCs on activation of Th17 cells.

### Aberrant effects of SAA MSCs on Treg differentiation

**Figure 4:**
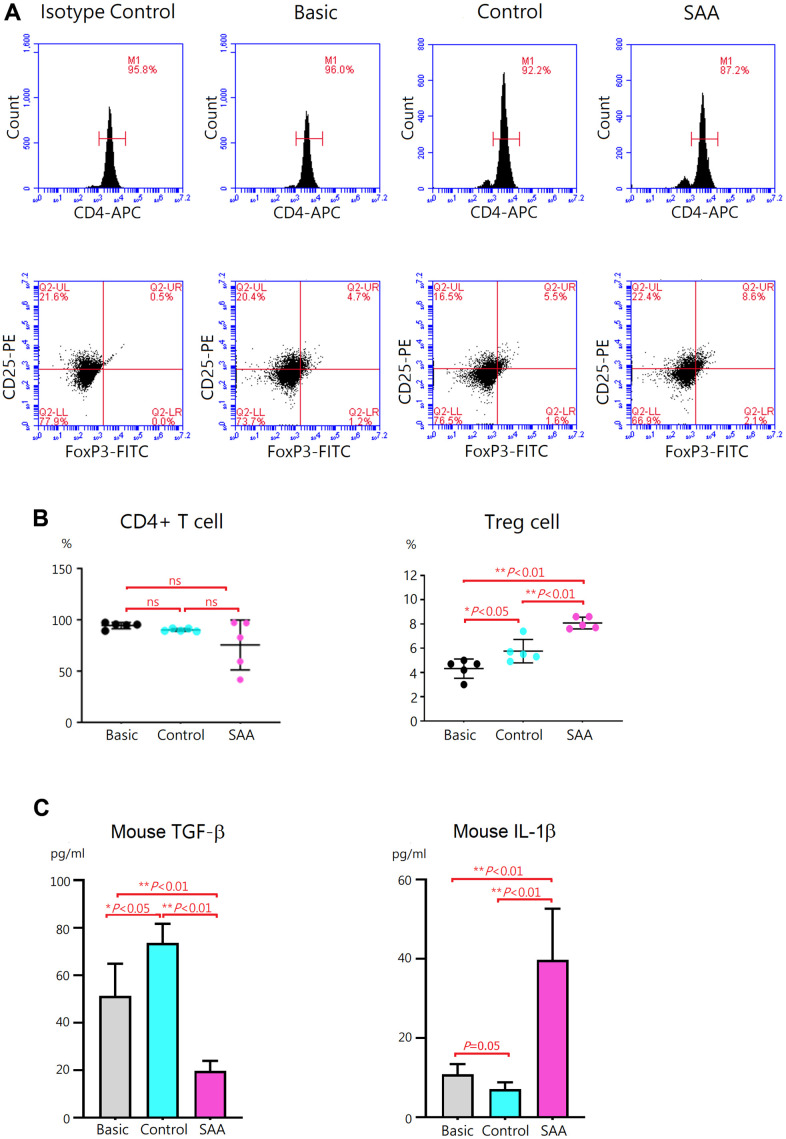
**Evaluation of Treg differentiation after 5-days differentiation induction.** (**A**) Differentiation of Treg cells was confirmed by intracellular staining for FoxP3 with flow cytometry. (**B**) The percentage of CD4+CD25+ FoxP3+ Treg cells was higher in the control group, compared to the basic group (5.8 ± 0.8% vs 4.3 ± 0.8%, *P* < 0.05). The percentage of CD4+CD25+ FoxP3+ Treg cells was significantly increased in the SAA group, compared to the control groups (8.1 ± 0.5% vs 5.8 ± 0.8%, *P* < 0.01). (**C**) In the SAA groups, TGF-β concentrations in the culture supernatant were decreased and IL-1β levels were increased, compared to the control group (*P* < 0.01). Data are presented as the mean ± SD. *n* = 5 in each group.

To evaluate effects of MSCs on regulatory T (Treg) differentiation, mouse CD4+ T cells were cocultured for 3 days with control MSCs or SAA MSCs after 48 hours Treg differentiation induction. At the end of differentiation on day 5, differentiation of CD4+ T cells into CD4+CD25+FoxP3+ Treg cells was confirmed by intracellular staining for FoxP3 by flow cytometry (Figure 4A). Compared to the basic group, no obvious alteration in the percentage of CD4+ T cells was observed either in the control group or SAA group after Treg differentiation induction, implicating that coculturing with MSCs did not affect the quantity of CD4+ T cell population (Figure 4B). Further analysis showed that a population of CD4+CD25+FoxP3+ Treg cells in the basic group was documented at the percentage of 4.3 ± 0.8% under 5-days Treg polarization conditions. In the control group, the percentage of Treg cells was significantly increased (5.8 ± 0.8%, *P* < 0.05). Consistently, transforming growth factor-β (TGF-β) concentrations in the cell culture supernatant were significantly increased and IL-1β levels were decreased, compared to the basic group (Figure 4C). These data indicated that coculture of CD4+ T cells with control MSCs could enhance the differentiation into CD4+CD25+FoxP3+ Treg cells under polarization conditions. Again, our results showed xenogeneic MSCs of human origin could have immunosuppressive effects on mouse immune cells. It is interesting to note that the percentage of Treg cells in the SAA group was significantly higher compared to the control group (8.1 ± 0.5% vs 5.8 ± 0.8%, *P* < 0.01), suggesting the promotive effect of SAA MSCs on Treg differentiation (Figure 4B). Inconsistent with the increased number of Treg cells, TGF-β concentrations in the culture supernatant were conversely decreased and IL-1β levels were elevated in the SAA group (Figure 4C). These results suggested that coculture of CD4+ T cells with SAA MSCs during differentiation into Treg cells may induce the production of more Treg cells but without adequate function.

## DISCUSSION

Immune-mediated destruction to HSCs is important for the pathogenesis of idiopathic SAA in children. MSCs are potent immune regulators and play a central role in providing the specialized bone marrow microenvironment for hematopoiesis. Dysregulation of immune cells from MSCs may lead to insufficiency of the bone marrow niche, which could be associated with hematopoietic impairment in SAA. In the present study, impact of SAA MSCs on T cell differentiation was evaluated by coculture of mouse CD4+ T cells with MSCs under Treg and Th17 polarization conditions. We found that inhibitory effects of SAA MSCs on differentiation of CD4+ T cells into Th17 cells were attenuated, as well as activation of Th17 cells. Aberrant effects of SAA MSCs on Treg differentiation were demonstrated by the production of dysfunctional Treg cells. Increased IL-17 and IL-1β levels along with decreased TGF-β levels in the culture supernatant suggested the potential of SAA MSCs for triggering a hyperinflammatory environment. The chaotic immunoregulation of SAA MSCs could be associated with the lack of their immunoprotection in the bone marrow and the development of SAA.

Within the bone marrow, MSCs are essential for the specialized niche, promoting maintenance, proliferation, and differentiation of HSCs. Using long-term culture system, deficiency in stromal layer formation and HSC maintenance was demonstrated in bone marrow stromal cells isolated from patients with SAA [14, 15]. We and other investigators examined hematopoiesis-supporting abilities of SAA MSCs, and found reduced proliferation of peripheral blood mononuclear cells and decreased colony forming capacity of CD34+ cells when cocultured with SAA MSCs [10, 16]. These data indicated the potential pathophysiological mechanism of MSC defects causing bone marrow failure in SAA.

To clarify how MSCs lose their ability to hematopoietic support, many studies have been conducted to examine individual characteristics and functions of SAA MSCs [17]. Using their basic properties as indicators, we firstly documented lower potential of proliferation and differentiation in SAA MSCs without changes in their morphologies and surface marker expressions [8]. Since then, these characteristics of SAA MSCs have been investigated in a number of studies [18]. The majority indicated worse proliferative ability of SAA MSCs, and all studies demonstrated no difference in their surface marker expression. Most studies showed a decreased propensity of SAA MSCs toward osteogenesis. But results of adipogenesis were quite discrepant, some reported an increased tendency of SAA MSCs to differentiate toward adipocytes [19–22] and some reported a decreased capacity [8, 23]. Despite the underlying reasons for the discrepancy remain to be elucidated, alterations of differentiation potential in SAA MSCs do exist. Several studies have been conducted to clarify mechanisms of poor proliferation in SAA MSCs. We and other researchers observed an increased rate of apoptosis in SAA MSCs and a higher percentage of cells in the abnormal sub-G1 phase of the cell cycle. SAA MSCs were prone to senescence, shown by a higher proportion of β-galactosidase staining cells and failure to passage [10, 21]. Consistent with these abnormal features, aberrant expression profiles of related genes in SAA MSCs were reported [9, 21, 24].

In addition to compromised hematopoiesis-supporting abilities of SAA MSCs, another important concept proposed is related to the lack of immunoprotection in SAA bone marrow. While immune responses are certainly complex, homeostatic dysregulation of the T cell repertoire has been implicated in HSC destruction in SAA bone marrow. It has been demonstrated that the number of activated CD8+ T cells is increased in both bone marrow and peripheral blood of patients with SAA. Coculture CD8+ T cells isolated from patients with SAA was found to inhibit colony formation and enhance apoptosis in CD34+ cells isolated from normal individuals [25–27], suggesting that CD8+ T cells in patients with SAA were dysfunctional. Abnormalities in the number and function of CD4+ T cells in patients with SAA have also been reported, with increased Th1, Th2, and Th17 cells, and decreased Treg cells [28]. Consistent with these findings, hyperfunctioning T cells may release a variety of inflammatory cytokines, such as interferon-γ (IFN-γ), tumor necrosis factor-α (TNF-α), and IL-17, thus with elevated concentrations in SAA bone marrow plasma [29–31]. These cytokines were found to have additional hematopoietic suppression by inducing programmed cell death of CD34+ cells [27].

With great immunomodulatory functions, MSCs are essential for the maintenance of immune homeostasis in the bone marrow by fine-tuning immune cell activities. Dysfunctional immunomodulation of MSCs may contribute to a hyperinflammatory status in the bone marrow, which could be associated with immune-mediated HSC destruction in SAA. However, limited data in the literature were available regarding this important issue. Using *in vitro* coculture systems, the reduced ability of SAA MSCs to inhibit proliferation and activation of T cells was demonstrated [21, 32]. The inhibitory defects of SAA MSCs influenced both CD4+ T cells and CD8+ T cells [21, 33]. SAA MSCs were also found to alter the differentiation capacity of CD4+ T cells. Previous studies consistently demonstrated that coculturing with SAA MSCs showed decreased inhibition on differentiation of CD4+ T cells into Th1 cells but no obvious effects on Th2 differentiation [21, 33]. However, there was discrepancy in the impact of SAA MSCs on Th17 differentiation, decreased inhibitory capacity in one report [21] but no obvious change in the other [33]. On the other hand, only one study reported defective ability of SAA MSCs in inducing Treg expansion [33]. Therefore, we aimed to examine whether SAA MSCs have alterant effects on Th17 and Treg differentiation in the present study. We found attenuated inhibition on differentiation of CD4+ T cells into Th17 cells when cocultured with SAA MSCs. There were aberrant effects of SAA MSCs on Treg differentiation, illustrating with increased Treg cells but significantly lower levels of TGF-β in the culture supernatant. As higher IL-1β levels in the culture supernatant, the influence of SAA MSCs on Treg differentiation made both quantitative and qualitative alterations, with the production of a greater amount of dysfunctional Treg cells.

In our previous study, aberrant cytokine profiles in the conditioned medium of SAA MSCs were found, with increased concentrations of IL-1β, IL-6, IFN-γ, and TNF-α [10]. To examine whether SAA MSCs exert alterant immunomodulation on Th17 and Treg cells, we compared the production of IL-1β, IL-17, and TGF-β by T cells in the presence of MSCs in the present study. After Th17 polarization, IL-1β and IL-17 levels in the culture supernatant were significantly higher when cocultured with SAA MSCs, compared to control MSCs. While TGF-β within the stimulation cocktail could confound the measurement of TGF-β secreted by T cells, we minimalized the inaccuracy by adding the same amount of cytokine cocktail in each well before differentiation. After Treg induction, higher IL-1β and lower TGF-β levels in the culture supernatant were detected when cocultured with SAA MSCs. These data implicated the potential of dysfunctional SAA MSCs for triggering a hyperinflammatory environment.

The beneficial effects of MSC therapy for patients with SAA have been demonstrated in many clinical reports [17]. The majority of patients received co-infusion of allogeneic MSCs during HSC transplantation and the most evident benefit was the improvement of engraftment [7, 34–37], implicating the poor ability of SAA MSCs to repopulate the bone marrow niche. The performance and efficacy of MSCs appears to be cross-species. Therefore, it is convenient and rational to investigate their action by using MSCs from different species for experiments. Xenogeneic MSCs of human origin have been used in numerous animal models of diseases, such as sepsis [38], acute lung injury [39], immune disorders [40], and aplastic anemia [41, 42]. In consideration of the availability and ethical issues, T cells isolated from mouse splenocytes were used as the effector cells to evaluate immunomodulatory functions of human MSCs in the present study.

The present study has several limitations. First, mix and xeno-culture of human MSCs and mouse T cells was used to investigate immunomodulatory effects of MSCs on T cell differentiation. Although MSC function may be maintained across species, using allogeneic origins of MSCs and HSCs in future studies to confirm this important issue is needed. Second, the present study only determined the influence of MSCs on differentiation of CD4+ T cells into Th17 and Treg cells. As MSCs can exert immunomodulatory function on a variety of immune cells, further studies including more induction conditions to provide a global view are encouraged.

## MATERIALS AND METHODS

### Patients

Idiopathic SAA was defined as pancytopenia and hypocellular bone marrow after excluding any other underlying diseases. The inclusion criteria were bone marrow hypocellularity of less than 25% and at least two of following: absolute neutrophil count < 0.5 × 10^9^ /l, platelet count < 20 × 10^9^ /l, and reticulocyte count < 1% [3]. Control subjects were patients who received bone marrow examination for diseases other than hematological diseases with pathological proof of normal bone marrow. All participants were younger than 18 years of age. This study was approved by the Institutional Review Board of the Chung Shan Medical University Hospital (CS2-21056), and written informed consents were obtained from the parents.

### MSC isolation

MSCs were collected, isolated, and identified as in our previous reports [8–10]. Bone marrow cells were collected from iliac crest aspirates, and mononuclear cells were isolated by Ficoll-Paque density centrifugation (1.077 g/ml; Amersham Biosciences, Uppsala, Sweden). Then, cells were seeded in low-glucose DMEM (Gibco, Gaithersburg, MD, USA) supplemented with 10% fetal bovine serum (FBS) and antibiotic/antimycotic, and were incubated at 37° C in a humidified atmosphere under 5% CO_2_. The medium with suspension of non-adhered cells was discarded after 48 hours, and replaced twice a week thereafter. Upon reaching 80-90% confluence, cells were detached with trypsin-EDTA (Gibco, Carlsbad, CA, USA) and re-plated for subculture. Under the approval of the institutional review board, parts of the cultured MSCs were stored at -80° C. The stored cells were thawed and further cultured, and MSCs of passage 5 were used for experiments in the present study.

### MSC identification

The criteria of International Society for Cellular Therapy were used to characterize MSCs [13]. To evaluate immunophenotypic expression, cultured MSCs were detached, washed, and resuspended in phosphate-buffered saline. After fixing and blocking, cells were immunolabeled with phycoerythrin (PE) mouse anti-human CD34 (Clone 8G12; BD Biosciences, San Jose, CA, USA), fluorescein isothiocyanate (FITC) mouse anti-human CD45 (Clone 2D1; BD Biosciences, San Jose, CA, USA), allophycocyanin (APC) mouse anti-human CD44 (Clone G44-26; BD Biosciences, San Jose, CA, USA), or PE mouse anti-human CD105 (Clone 266; BD Biosciences, San Jose, CA, USA) antibodies. Mouse IgG (BD Biosciences, San Jose, CA, USA) served as isotype control. Data were analyzed by flow cytometry (Accuri C6; BD Biosciences, San Jose, CA, USA). To assess differentiation potential, cultured MSCs were detached and replated in 60-mm dishes. For osteogenic induction, MSCs were grown in DMEM with 10% FBS, 10 mM β-glycerophosphate, 0.1 μM dexamethasone, and 0.2 mM ascorbic acid. For adipogenic induction, MSCs were grown in DMEM with 10% FBS, 1 μM dexamethasone, 0.5 mM 3-isobutyl-1-methylxanthine, 0.1 mM indomethacin, and 10 μg/ml insulin. After 2-week induction, osteogenesis and adipogenesis were demonstrated by von Kossa stain (Cedarlane, Ontario, Canada) and oil red O stain (Sigma, St Louis, MO, USA), respectively.

### Preparation of CD4+ T cells from mice

Eight-week-old C57BL/6 mice were purchased from the National Laboratory Animal Center. The experimental protocol was approved by the Institutional Animal Care and Use Committee of the Chung Shan Medical University Experimental Animal Center (IACUC Approval No: 2488). Immediately after sacrifices, spleen tissue samples were obtained, ground, and passed through 40 μm strainer. MojoSort Mouse CD4 T Cell Isolation Kit (BioLegend, San Diego, CA, USA) was used for purification of the collected CD4+ cells by negative selection, according to the manufacturer’s instruction. The purity of CD4+ cells was measured with the Accuri C6 flow cytometer. The final purified cells were collected and cultured in RPMI-1640 (Gibco, Waltham, MA, USA) supplemented with 10% FBS and antibiotic/antimycotic. These cells were used as CD4+ T cells for the following studies.

### Coculture of CD4+ T cells with MSCs during Th17 differentiation induction

CellXVivo Mouse Th17 Cell Differentiation Kit (R&D Systems, Minneapolis, MN, USA) was used to induce differentiation of Th17 cells from the preparation of CD4+ T cells isolated from mouse splenocytes, according to the manufacturer’s instruction. CD4+ T cells were differentiated for a total of 5 days under Th17 polarization conditions. To assess effects of MSCs on Th17 differentiation, CD4+ T cells were cocultured with control MSCs or SAA MSCs in 24-well dishes without physical separation after 48 hours of differentiation induction. The ratio of CD4+ T cells to MSCs was 100:1. In the coculture system, MSCs could interact with CD4+ T cells through both direct cell-to-cell contact and paracrine mechanisms. For baseline control, CD4+ T cells in the basic group were differentiated for 5 days under the same conditions but without coculturing with MSCs. At the end of differentiation on day 5, the cell culture supernatant was collected and cytokine secretion was determined using the Mouse IL-17A ELISA kit (BioLegend, San Diego, CA, USA) and the Mouse IL-1β ELISA kit (BioLegend, San Diego, CA, USA). The levels were determined in triplicate.

### Assessment of Th17 activation

After a 5-days Th17 differentiation, the cells were stimulated with the Cell Activation Cocktail with Brefeldin A (BioLegend, San Diego, CA, USA) for 6 hours. Following activation of cells with this cocktail, the cell culture supernatant was collected and concentrations of IL-17 and IL-1β were measured by ELISA as previously described. For intracellular detection of the secreted cytokines, the activated cells were stained with APC anti-mouse CD4 antibody (Clone GK1.5; BioLegend, San Diego, CA, USA) and then treated with Intracellular Fixation and Permeabilization Buffer Set and FITC anti-mouse IL-17A antibody (Clone TC11-18H10.1; BioLegend, San Diego, CA, USA). Thereafter, the cells were examined by the Accuri C6 flow cytometer.

### Coculture of CD4+ T cells with MSCs during Treg differentiation induction

CellXVivo Mouse Treg Cell Differentiation Kit (R&D Systems, Minneapolis, MN, USA) was used to induce differentiation of Treg cells from the preparation of CD4+ T cells isolated from mouse splenocytes, according to the manufacturer’s instruction. There was a total of 5 days for Treg differentiation induction. To evaluate influences of MSCs on differentiation of CD4+ T cells into Treg cells, CD4+ T cells were cocultured with control MSCs or SAA MSCs at the ratio of 100:1 after 48 hours of differentiation induction. As the procedure of Th17 polarization, MSCs could interact with CD4+ T cells in the coculture system through both direct cell-to-cell contact and secreting soluble factors. For baseline control, CD4+ T cells in the basic group were differentiated for 5 days under the same conditions but without coculturing with MSCs. On day 5 of differentiation, the cell culture supernatant was collected and cytokine secretion was determined using the TGF-β1 Mouse ELISA kit (ThermoFisher Scientific, Waltham, MA, USA) and the Mouse IL-1β ELISA kit. The levels were determined in triplicate.

Treg cell differentiation was further documented by analyzing marker expression via flow cytometry. The differentiated cells were stained with APC anti-mouse CD4 antibody and PE anti-mouse CD25 antibody (Clone 3C7; BioLegend, San Diego, CA, USA), followed by fixation and permeabilization with FOXP3 Fix/Perm Buffer Set (BioLegend, San Diego, CA, USA). Then, the cells were stained with FITC anti-mouse FOXP3 antibody (Clone 150D; BioLegend, San Diego, CA, USA) and analyzed by the Accuri C6 flow cytometer.

### Statistical analysis

Data analysis was performed using GraphPad Prism 9.0 software. For continuous variables, Mann-Whitney U test was used to compare groups. A value of *P* < 0.05 was considered to be statistically significant.

## Supplementary Material

Supplementary Figures
