# Supplementary material for: Alterations of mesenchymal stem cells on regulating Th17 and Treg differentiation in severe aplastic anemia
Source: Aging (Albany NY). 2023 Jan 30;15(2):553–66. doi: 10.18632/aging.204500 (PMC9925683; doi:10.18632/aging.204500)
Supplement: Supplementary Figures [file aging-15-204500-s001.pdf]

SUPPLEMENTARY FIGURES

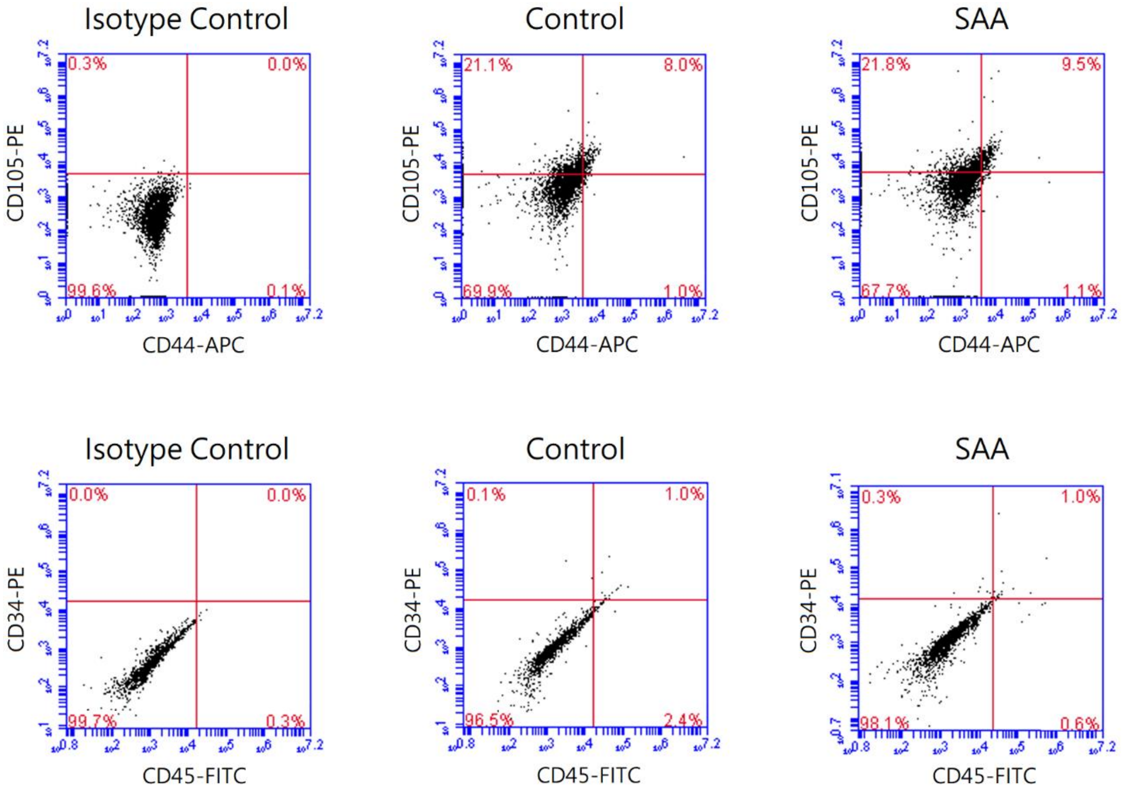

Supplementary Figure 1. Characterization of MSCs using flow cytometry. MSCs were stained with CD34-PE, CD45-FITC, CD44-APC, and CD105-PE, and analyzed using flow cytometry. Expression of these surface markers was illustrated in scatter plots and quantified results.

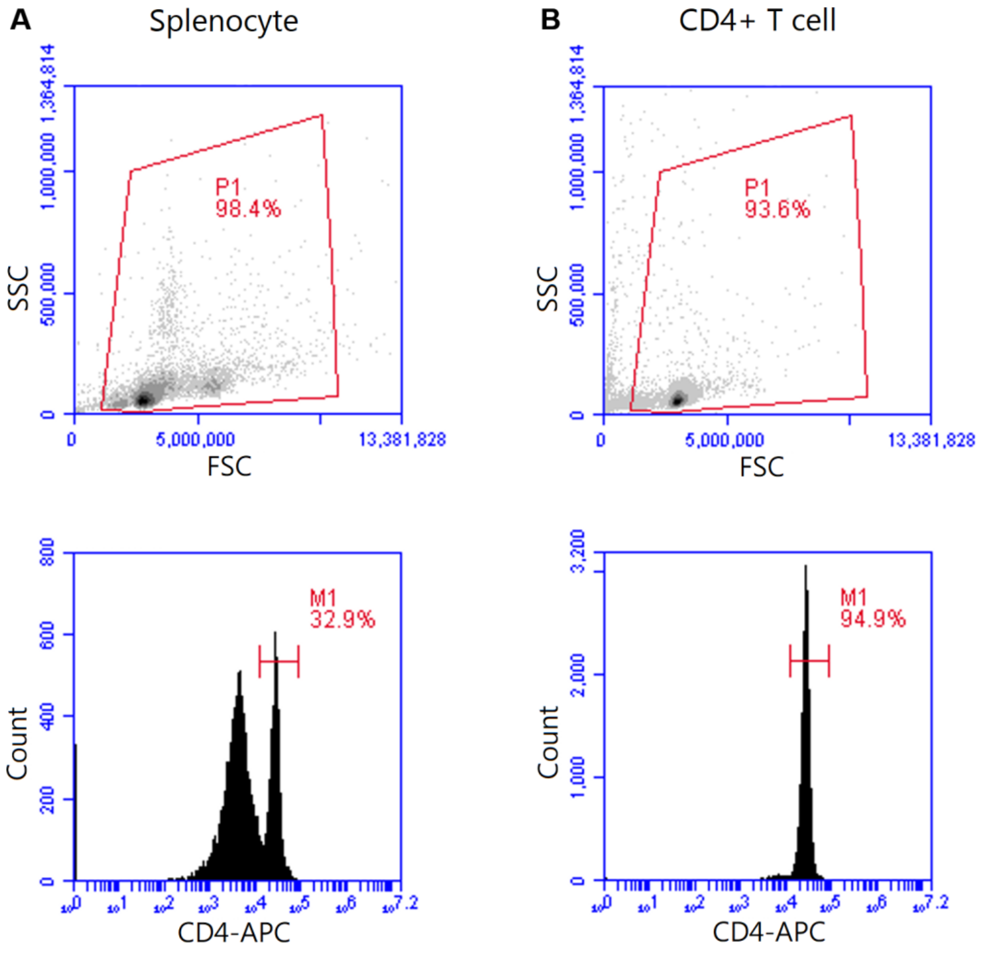

Supplementary Figure 2. Isolation and purification of CD4+ T cells from splenocytes of C57BL/6 mice using the negative selection protocol. (**A**) Using flow cytometry analysis, about 32.9% of splenocytes were CD4+ cells. (**B**) Purified CD4+ cells were further isolated by negative selection, and the final purity was 94.9%.

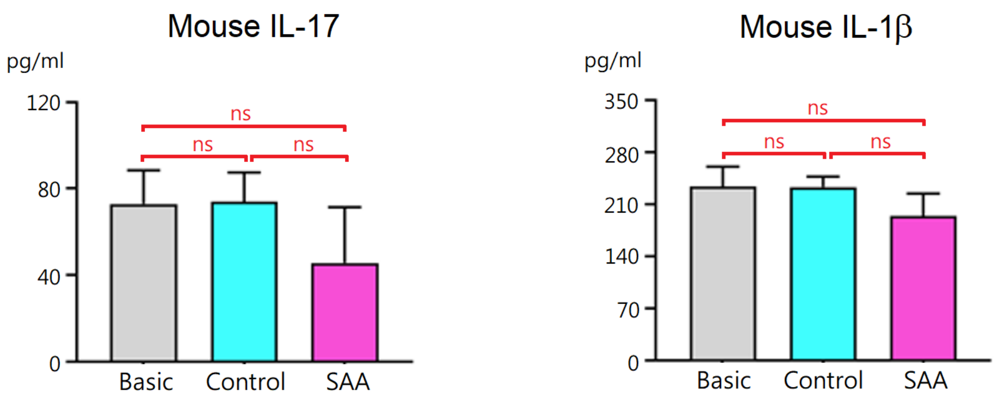

Supplementary Figure 3. Assessment of Th17 activation after 5-days Th17 differentiation. There were no differences in concentrations of mouse IL-17 and IL-1β in the supernatant in the basic, control, and SAA groups.
